# Synthesis and Application of Thiourea–Poly(Acrylic Acid)–Formaldehyde Composites for Removal of Crystal Violet Dye

**DOI:** 10.3390/ma18235462

**Published:** 2025-12-04

**Authors:** Adel Elamri, Khmais Zdiri, Kamila Bourkaib, Mahjoub Jabli, Adnane Labed, Sophie Bistac, Omar Anis Harzallah

**Affiliations:** 1 Textile Materials and Processes Laboratory (MPTex), National Engineering School of Monastir, University of Monastir, Monastir 5019, Tunisia; khmaiszdiri@gmail.com; 2 Centre de Recherche en Sciences et Technologie de Textile à Monastir (CRSTTM), Université de Monastir, Monastir 5019, Tunisia; 3 Laboratory of Functional Analysis of Chemical Processes, Faculty of Technology, Blida1 University, Blida 09000, Algeria; bourkaibcamila@gmail.com; 4 Department of Chemistry, College of Science, Majmaah University, Al-Majmaah 11952, Saudi Arabia; 5 Laboratory of Mechanical Engineering (LGM), University of Biskra, B.P. 145 R.P., Biskra 07000, Algeria; a.labed@univ-biskra.dz; 6 Laboratoire de Photochimie et d’Ingenierie Macromoleculaires (LPIM EA 4567), Ecole Nationale Supérieure de Chimie de Mulhouse (ENSCM), Université de Haute Alsace, 68100 Mulhouse, France; sophie.bistac-brogly@uha.fr; 7 Laboratoire de Physique et Mécanique Textiles (LPMT UR 4365), University of Haute-Alsace (UHA), 68093 Mulhouse, France

**Keywords:** thiourea–formaldehyde, poly(acrylic acid), composites, dye, adsorption, crystal violet

## Abstract

Textile dye effluents, particularly cationic dyes, pose a major environmental challenge, demanding efficient and sustainable adsorbent materials to remove harmful synthetic dyes. In this study, a reference thiourea–formaldehyde (TU/FA) composite and a series of thiourea–poly(acrylic acid)–formaldehyde (TU/PAA/FA) composites were synthesized and systematically characterized. The composites were prepared by varying the volume of poly(acrylic acid) PAA (from 1 to 7.5 mL) to assess how PAA incorporation influences morphology, crystallinity, surface chemistry, charge, and thermal stability. Analytical techniques including SEM, XRD, FT-IR, particle size distribution, zeta potential, and TGA/DTG revealed that increasing PAA content induced more porous and amorphous microstructures, intensified carbonyl absorption, reduced particle size (optimal at 2.5–5 mL PAA), and shifted the zeta potential from near-neutral to highly negative values (−37 to −41 mV). From TU/PAA/FA composite analysis, it was depicted that the TU/PAA-5/FA material has the better characteristics as a potential cationic dye absorbent. Thus, the adsorption performance of this composite toward crystal violet dye was subsequently investigated and compared to the reference material thiourea–formaldehyde (TU/FA). The TU/PAA-5/FA material exhibited the highest capacity (145 mg/g), nearly twice that of TU/FA (74 mg/g), due to the higher density of carboxylic groups facilitating electrostatic attraction. Adsorption was pH-dependent, maximized at pH 6, and decreased with temperature, confirming an exothermic process. Kinetic data followed a pseudo-second-order model (R^2^ = 0.99), implying chemisorption as the rate-limiting step, while Langmuir isotherms (R^2^ > 0.97) indicated monolayer adsorption. Thermodynamic analysis (ΔH° < 0, ΔS° < 0, ΔG° > 0) further supported an exothermic, non-spontaneous mechanism. Overall, the TU/PAA-5/FA composite combines enhanced structural stability with high adsorption efficiency, highlighting its potential as a promising, low-cost material for the removal of cationic dyes from textile effluents.

## 1. Introduction

Water contamination by textile dyes represents a major global environmental and public health challenge. These pollutants are often chemically stable and persistent, highly visible even at low concentrations, and pose toxic and carcinogenic risks to aquatic organisms and humans [1]. Effluents from the textile industry therefore constitute a critical source of organic contamination, needing the development of treatment technologies that are efficient, simple, and economically viable [2]. Cationic dyes such as crystal violet (CV) are extensively used in the textile, paper, and leather industries and are known for their mutagenic and carcinogenic effects, as well as for their persistence in wastewater. Therefore, the development of efficient, low-cost, and environmentally friendly adsorbents capable of removing such pollutants has become an urgent concern.

Among the various remediation strategies, adsorption has emerged as a particularly attractive approach due to its operational simplicity, low cost, and effectiveness, even at low concentrations of cationic dyes [3]. The performance of an adsorbent, however, depends on key structural and chemical features such as the density and accessibility of active sites, porosity, microstructure, and surface chemistry. In this context, material engineering plays a crucial role in optimizing adsorption properties [4].

Polymeric materials and composites functionalized with acidic or chelating groups have attracted increasing attention for the removal of cationic dyes, including Methylene Blue, crystal violet, and Malachite Green synthetic dyes [5]. Carboxylate and sulfonate groups on the adsorbent surface provide negatively charged sites that promote electrostatic interactions with dye molecules. Consequently, controlling microstructure and surface charge is a decisive factor in the design of effective adsorbents [6].

Thiourea-based composites, particularly thiourea–formaldehyde (TU/FA) networks, contain heteroatoms (nitrogen and sulfur) capable of forming hydrogen bonds and coordination interactions. These materials have been successfully employed as functional modifiers in various remediation systems to improve the adsorption of pollutants including heavy metals and some dyes [7,8]. Poly(acrylic acid) (PAA) introduces carboxylic groups that allow simultaneous modulation of porosity, surface charge, and hydrophilicity when incorporated into composites [9].

Motivated by these complementary features, we developed a series of TU/PAA/FA composites by pre-mixing aqueous PAA with thiourea prior to formaldehyde-induced polycondensation. This pre-mixing strategy is intended to influence nucleation, growth, and crosslinking stages, thereby controlling microstructure and functional site accessibility [10]. Incorporating PAA also enables tuning of the morphology, particle size distribution, surface charge, and thermal stability of the composites [11].

In this context, TU/FA composites serve as an ideal matrix: they are rich in heteroatoms (N, S), facilitate hydrogen bonding and coordination with adsorbed molecules, and exhibit good chemical and thermal stability [12]. The addition of PAA provides extra carboxylic functionalities, modulating surface charge, porosity, and hydrophilicity, while formaldehyde ensures network crosslinking. This combination allows the design of composites with optimized physicochemical properties for cationic dye adsorption [13,14].

The present work reports the synthesis and multi-technique characterization of TU/PAA/FA composites, including scanning electron microscopy (SEM), X-ray diffraction (XRD), Fourier-transform infrared spectroscopy (FTIR), particle size analysis, and zeta potential and conductivity measurements, as well as thermal studies using thermogravimetric analysis and derivative thermogravimetry (TGA/DTG). The relationships between PAA content, structure, and physicochemical properties relevant to adsorption are established.

Beyond the structural and physicochemical characterization, the practical relevance of the developed TU/PAA/FA composites was evaluated through their application in the adsorption of crystal violet (CV), a representative cationic dye widely used in the textile industry and known for its toxicity and persistence in aquatic environments. The adsorption kinetics were modeled using pseudo-first-order, pseudo-second-order, Elovich, and intraparticle diffusion equations to identify the rate-limiting steps and underlying mechanisms. Thermodynamic parameters (ΔH°, ΔS°, ΔG°) were also determined to better understand the nature of the dye–adsorbent interaction.

## 2. Materials and Methods

### 2.1. Materials

Thiourea (TU, reagent-grade) was selected for its ability to react with formaldehyde and form polymeric networks rich in sulfur-containing functional groups, enabling the preparation of composites with stable structures. Formaldehyde solution (FA, typically 37 wt% aqueous formalin) was employed as a crosslinking agent to promote covalent bonding between thiourea and/or poly(acrylic acid) chains, ensuring cohesion and thermal resistance of the final material.

Poly(acrylic acid) (PAA, MW = 250 kDa, 35% *w*/*w* solution) was incorporated to introduce hydrophilic carboxylic groups, enhancing water compatibility and modulating the mechanical and thermal properties of the composites.

Crystal violet (C_25_H_30_ClN_3_, MW = 407.98 g/mol, λ_max_ in water = 590 nm) was used as the adsorbate and the colored solutions were prepared using distilled water.

Thiourea, formaldehyde, poly(acrylic acid), and crystal violet were purchased from Sigma-Aldrich (Dorset, UK). The pH of the aqueous solutions was adjusted using either HCl (0.1 M) or NaOH (0.1 M) solutions.

**Scheme 1:**
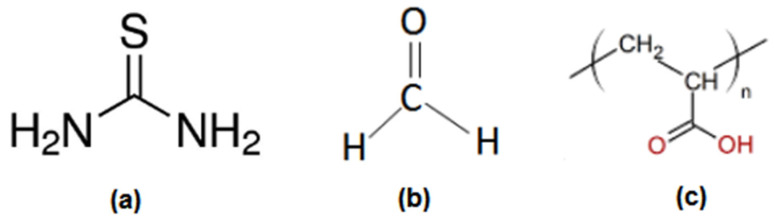
Chemical formulas of (**a**) thiourea, (**b**) formaldehyde, and (**c**) PAA.

The chemical formulas of the used products are reported in Scheme 1.

### 2.2. Synthesis of Composites

The experimental protocol was designed to produce thiourea–formaldehyde composites (TU/FA) as a reference, and thiourea–poly(acrylic acid)–formaldehyde composites (TU/PAA/FA) by varying the incorporated PAA volume. The primary objective was first to examine the effect of pre-mixing PAA with thiourea before formaldehyde addition, and then to assess the influence of different PAA contents on composite properties.

#### 2.2.1. Preparation of TU/FA Composite

The reference TU/FA composite was prepared by dissolving 5.87 g of thiourea in 100 mL of distilled water, followed by the addition of 6 mL of formaldehyde. The mixture was stirred and maintained at 80 °C until precipitation occurred. The resulting precipitate was thoroughly washed with distilled water and dried at 60 °C. This reference composite was used for comparison with PAA-containing composites.

#### 2.2.2. Preparation of TU/PAA/FA Composites

For TU/PAA/FA composites, 5.87 g of thiourea was dissolved in 100 mL of distilled water, followed by the addition of varying volumes of PAA (1, 2.5, 5, and 7.5 mL). Each mixture was stirred at 80 °C to ensure homogeneity. Subsequently, 6 mL of formaldehyde was added gradually over 2 h at 80 °C until precipitation occurred. The composites were designated according to the PAA volume: TU/PAA-1/FA, TU/PAA-2.5/FA, TU/PAA-5/FA, and TU/PAA-7.5/FA. After formation, the precipitates were washed thoroughly with distilled water and dried at 60 °C.

Table 1 summarizes the composition and designations of the prepared composites, enabling evaluation of (i) the effect of pre-mixing PAA with thiourea before FA addition, and (ii) the influence of PAA content on structural and functional properties, using TU/FA as a reference.

### 2.3. Characterization Techniques

#### 2.3.1. Scanning Electron Microscopy

The morphology and microstructure of TU/FA and TU/PAA/FA composites were examined by scanning electron microscopy (SEM) in secondary electron mode. A few milligrams of each composite powder was mounted on a sample holder using adhesive carbon tape and coated with a thin conductive layer of gold. Observations were performed using a Scanning Electron Microscope JEOL-JSM 100 LT instrument (Tokyo, Japan), and micrographs were processed and analyzed using SigmaScan Pro 5.0 software.

#### 2.3.2. X-Ray Diffraction

X-ray diffraction (XRD) was used to characterize the crystalline structure of the composites. Several milligrams of powder were spread evenly on a flat sample holder. Diffractograms were recorded using a diffractometer with a Cu Kα source (λ = 1.5406 Å) over a 2θ range suitable for polymeric materials. This allowed identification of diffraction peaks associated with crystalline organization and estimation of crystallinity. Diffractograms were analyzed using EVA-V6 (Bruker, Zurich, Switzerland)) software by comparing experimental data with reference databases to identify crystalline phases.

#### 2.3.3. Fourier-Transform Infrared Spectroscopy (FT-IR)

FT-IR analysis was performed to identify functional groups and assess chemical interactions within TU/FA and TU/PAA/FA composites. Measurements were conducted in attenuated total reflectance (ATR) mode over 4000–400 cm^−1^ using a PerkinElmer 783 IR (PerkinElmer, Hopkinton, MA, USA) spectrometer equipped with a single-bounce diamond ATR Smart Endurance cell. Each spectrum was acquired with 64 co-added scans at a resolution of 4 cm^−1^ and a mirror speed of 0.63 cm/s.

#### 2.3.4. Light Scattering (LS)

Particle size distributions were determined by light scattering (Laser Diffraction) for both number- and volume-based distributions. Characteristic parameters D10, D50, and D90 (representing 10%, 50%, and 90% of the cumulative particle volume) and the Sauter mean diameter (D32, indicative of surface-to-volume ratio) were calculated to characterize the granulometric distribution.

#### 2.3.5. Zeta Potential and Conductivity

The zeta potential (ZP) and electrical conductivity of polymer powder suspensions were measured using a Malvern Zetasizer (Malvern Panalytical, Malvern, UK). Measurements were conducted at room temperature (25 °C) using electrophoretic light scattering for ζ potential and integrated conductimetry for conductivity. All analyses were performed in triplicate, with results reported as mean ± standard deviation. Derived parameters, such as electrophoretic mobility (µm·cm/V·s), were automatically calculated by the instrument software.

#### 2.3.6. TGA/DTG

Thermogravimetric analysis (TGA) coupled with derivative thermogravimetry (DTG) was conducted using a TA Instruments Q500 thermobalance (TA Instruments, Guyancourt, France) controlled by Trios V5.1 software. Samples (8.0 ± 0.5 mg) were placed in alumina pans and heated from 25 to 600 °C under ultra-pure nitrogen at 10 °C/min. Prior to analysis, samples were equilibrated for 24 h at 25 °C under dry conditions. TGA and DTG curves were recorded simultaneously, and key parameters extracted included onset degradation temperature (T_onset), maximum degradation temperature (T_max), and residual carbon at 600 °C.

### 2.4. Batch Adsorption Study

An amount of 0.01 g of adsorbent and 20 mL of crystal violet solution were mixed in Erlenmeyer flasks and stirred at constant agitation (100 rpm). A variety of factors were investigated in order to optimize system parameters. The effect of pH ranges from 3 to 10 was assessed, and the pH was adjusted by utilizing 0.1 M HCl or 0.1 M NaOH. The samples were collected at regular intervals, with the contact time ranging from 0 to 120 min. The initial crystal violet concentration varied from 0 to 80 mg/L. Through the utilization of a temperature-controlled agitator, the temperature was changed from 22 °C to 45 °C. A UV–visible spectrophotometer Optizen 2120UV (Mecasys, Co. Ltd., Daejeon, Republic of Korea) and a quartz cuvette were employed to ascertain the residual dye concentration in the supernatant. The adsorbed dye capacity was then calculated.

The kinetic data was investigated using the common theoretical kinetic equations including pseudo-first-order, pseudo-second-order, Elovich, and intraparticle diffusion [15]. Their linear forms were given in the following equations:

(1) $$\log(q_{e}-q_{t})=\log q_{e}-K_{1}t$$

(2) $$\frac{t}{q_{t}}=\frac{1}{K_{2}{q_{e}}^{2}}+\frac{t}{q_{e}}$$

(3) $$q_{t}=\frac{1}{\beta }Ln\left(\alpha \beta \right)+\frac{1}{\beta }Lnt$$

(4) $$q_{t}=k_{i}.t^{1/2}$$

where q_e_ is the adsorbed capacity (mg g^−1^) at equilibrium, q_t_, is the adsorbed capacity (mg g^−1^) at time t, k_1_ is the rate constant for pseudo-first-order (L min^−1^). k_2_ is the rate constant for pseudo-second-order (g mg^−1^ min^−1^), *α* (mg g^−1^ min^−1^) and *β* (mg g^−1^ min^−1^) are the constants of Elovich, and k_1_ is the rate constant for intraparticle diffusion.

However, the theoretical isotherm equations of Langmuir, Freundlich, and Temkin [16] are used to better evaluate the interaction between crystal violet, TU/FA, and TU/PAA-5/FA. The linear forms of these isotherms were given in the following equations:

(5) $$\frac{c_{e}}{q_{e}}=\frac{1}{q_{m.L}K_{L}}+\frac{c_{e}}{q_{m.L}}$$

(6) $$\ln q_{e}=\ln K_{F}+\left(\frac{1}{n}\right)L\ln c_{e}$$

(7) $$q_{e}=B_{T}Ln(A_{T})+B_{T}Ln(C_{e})$$

q_e_ is the adsorbed amount of the adsorbate (mg g^−1^), C_e_ is the remaining concentration (mg L^−1^), K_l_ is the Langmuir constant (L mg^−1^), n is the heterogeneous factor of Freundlich, K_F_ is the capacity of adsorption of Langmuir [mg g^−1^ (L mg^−1^)1/n], and B_t_ and A_t_ are Temkin constants.

## 3. Results

### 3.1. Morphology

**Figure 1:**
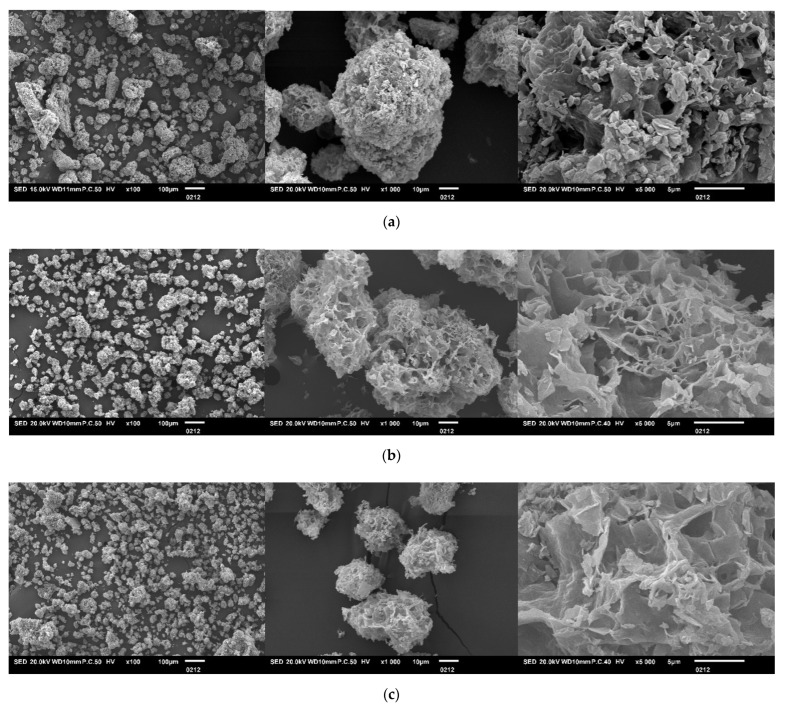

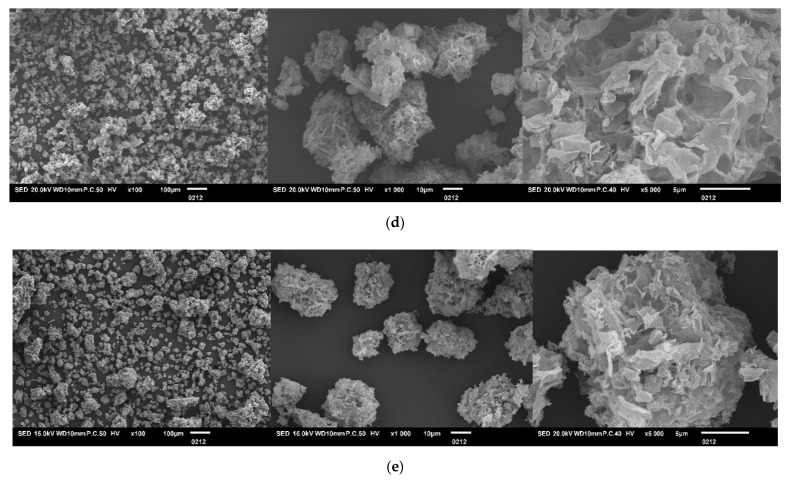
SEM micrographs of: (**a**) TU/FA, (**b**) TU/PAA-1/FA, (**c**) TU/PAA-2.5/FA, (**d**) TU/PAA-5/FA, (**e**) TU/PAA-7.5/FA composites at three different magnifications (from left to right: ×100, ×1000 and ×5000).

Scanning electron microscopy (SEM) images (Figure 1) clearly demonstrate the decisive influence of poly(acrylic acid) (PAA) and its incorporation sequence on the morphology of the prepared composites. SEM analysis showed that the reference TU/FA particles appear as small, irregular, and slightly agglomerated spheres. The compact and smooth spheres suggest that thiourea and formaldehyde precipitated concurrently in a homogeneous network. The resulting particles are dense, minimally porous, and form a compact structure.

Then, introducing PAA prior to formaldehyde markedly alters the morphology. Pre-mixing PAA reinforces the thiourea network and promotes the formation of a rougher, more porous surface. Carboxylic groups (–COOH) from PAA interact with amino and thiocarbonyl groups (–NH_2_ and –CSNH_2_) of thiourea through hydrogen bonding and potentially ionic interactions. This reinforcement stabilizes the polymer network and modifies the electronic environment of reactive sites. Subsequent formaldehyde addition reacts with available amino groups and some PAA carboxyl groups, forming a crosslinked network [17]. Pre-existing interactions between TU and PAA guide condensation, resulting in a more robust and interconnected structure.

Note that the gradually increasing PAA volume further influences the final morphology. At low PAA concentrations (1–2.5 mL), particles exhibit slight surface roughness with the emergence of initial superficial pores. Even a small amount of PAA promotes a more open structure through interactions between PAA –COOH groups and TU –NH groups, slightly improving surface area and adsorption potential.

For TU/PAA-5/FA, particles become highly porous and fragmented, with rough surfaces and complex microstructures. PAA intensifies structural expansion, generating deeper pores and a highly irregular surface. This trend is supported by slight shifts in –NH and –CO vibration bands observed in FTIR spectra, confirming that PAA directly affects porosity, specific surface area, and adsorption capacity. This morphology enhances diffusion and adsorption of cationic molecules.

With higher PAA content (TU/PAA-7.5/FA), particles display the most open and fragmented morphology, characterized by loose aggregates and a highly irregular, hollow surface. Enhanced PAA content promotes TU–PAA interactions, yielding a more porous and irregular SEM structure.

### 3.2. Structural Analysis

**Figure 2:**
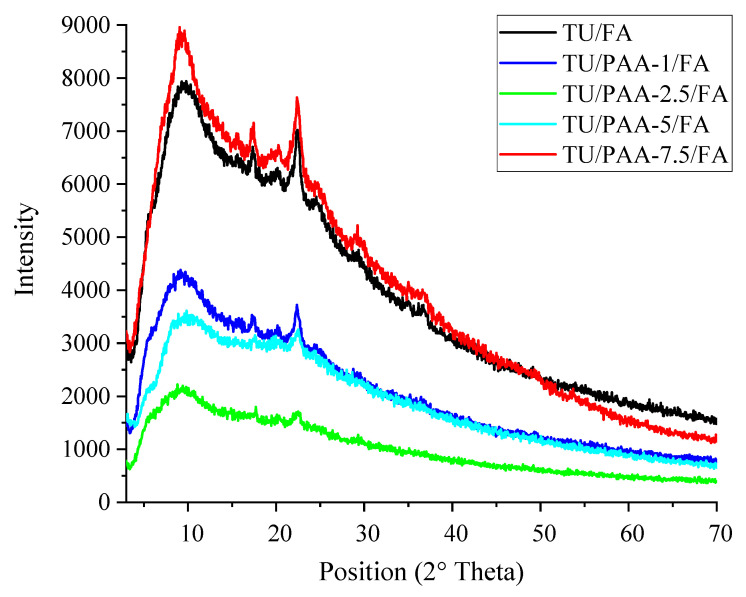
XRD patterns of TU/FA and TU/PAA/FA composites.

X-ray diffraction (XRD) patterns of TU/FA and TU/PAA/FA composites (Figure 2) reveal notable differences in crystalline structure depending on the sequence of PAA addition and the incorporated PAA volume. The TU/FA spectrum exhibits relatively intense and sharp peaks, indicating a partially crystalline structure. This crystallinity arises from the regular organization of thiourea–formaldehyde units [18]. Pre-incorporation of PAA into the thiourea solution prior to formaldehyde addition significantly alters the diffractograms compared to the TU/FA reference. The PAA–TU mixture promotes increased amorphization (weaker and broadened peaks) for low to medium PAA concentrations (1–5 mL), suggesting that PAA–TU interactions disrupt the nucleation and growth of crystalline domains typical of TU/FA. In contrast, at the highest PAA content (7.5 mL), a relative increase in peak intensity is observed, indicating partial reorganization or the formation of new polymeric aggregates.

Overall, PAA incorporation decreases composite crystallinity, favoring an amorphous structure. For low PAA volumes (TU/PAA-1/FA and TU/PAA-2.5/FA), a significant reduction in peak intensity relative to TU/FA is observed, indicating disruption of the original crystalline network due to interactions between PAA chains and the TU/FA matrix. This increased amorphization suggests better dispersion of polymer chains within the network. For intermediate PAA content (TU/PAA-5/FA), peaks remain attenuated, confirming a predominantly amorphous structure. At high PAA content (TU/PAA-7.5/FA), peak intensity rises again relative to other PAA-containing composites, suggesting partial structural reorganization and recrystallization induced by excess PAA [19]. These results highlight the key role of PAA in controlling the structural properties of TU/PAA/FA composites.

### 3.3. Functional Group Analysis

**Figure 3:**
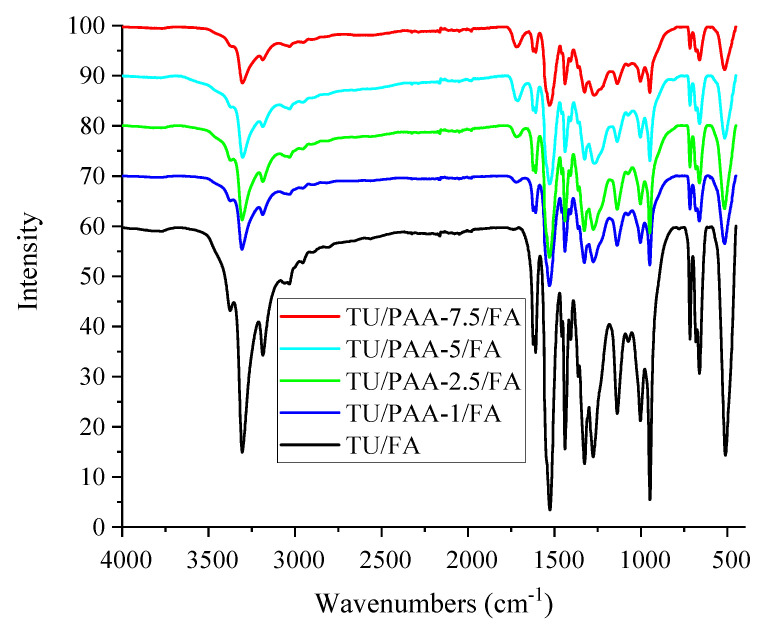
Infrared spectra of TU/FA and TU/PAA/FA composites.

The TU/FA reference spectrum (Figure 3) displays sharp and well-defined bands across multiple regions, indicative of a relatively structured material. These bands serve as a baseline for assessing the effects of PAA addition. The analysis of FTIR spectra of developed TU/PAA/FA composites showed the following main spectral regions [20,21,22]:

- 3500–3200 cm^−1^ (O–H/N–H): PAA-containing samples exhibit broadening, shifts, and partial intensity reduction relative to TU/FA, reflecting hydrogen bonding between PAA –COOH/–OH groups and thiourea –NH groups. The pre-mixing of PAA with thiourea before FA addition results in shifting and broadening of O–H/N–H bands (3500–3200 cm^−1^), confirming hydrogen bond formation between PAA and thiourea.
- This is also confirmed by the gradual emergence and intensification of the 1720–1700 cm^−1^ (C=O, PAA carboxylic) band as PAA content increases.
- ~1650–1550 cm^−1^ (N–H scissoring, C=N, possible amides): Observed shifts suggest the formation of new bonds or modifications in the chemical environment, likely associated with partial condensation between TU and FA.
- 3000–2850 cm^−1^ (C–H, CH_2_): Variations in this region indicate potential formation of methylene bridges via crosslinking with formaldehyde.
- 1300–1000 cm^−1^ (C–O/fingerprint, C–N/C–S): Changes in shape and intensity reflect either increased PAA incorporation or modifications of the C–N/C–S bonds in thiourea.
- <900–700 cm^−1^ (C–S/thiocarbonyl): Alterations may indicate changes in the thiocarbonyl environment.

FTIR analysis confirms that PAA is intimately integrated into the network, promoting better dispersion and enhanced inter-chain interactions. Spectroscopically, this manifests as broader bands and shifts rather than a simple superposition of TU and PAA signals.

The progressive increase in PAA content incorporated with thiourea leads to a more disordered chemical environment and increased hydrogen bonding, resulting in a more amorphous structure. In addition, high PAA content can stabilize specific sites via hydrogen bonding or direct methylene bridge formation differently, reflected in variations in band intensity and shape. Overall, FTIR profiles indicate a transition toward a more amorphous composite structure dominated by PAA–TU interactions rather than a mere physical mixture.

### 3.4. Particle Size Distribution

**Figure 4:**
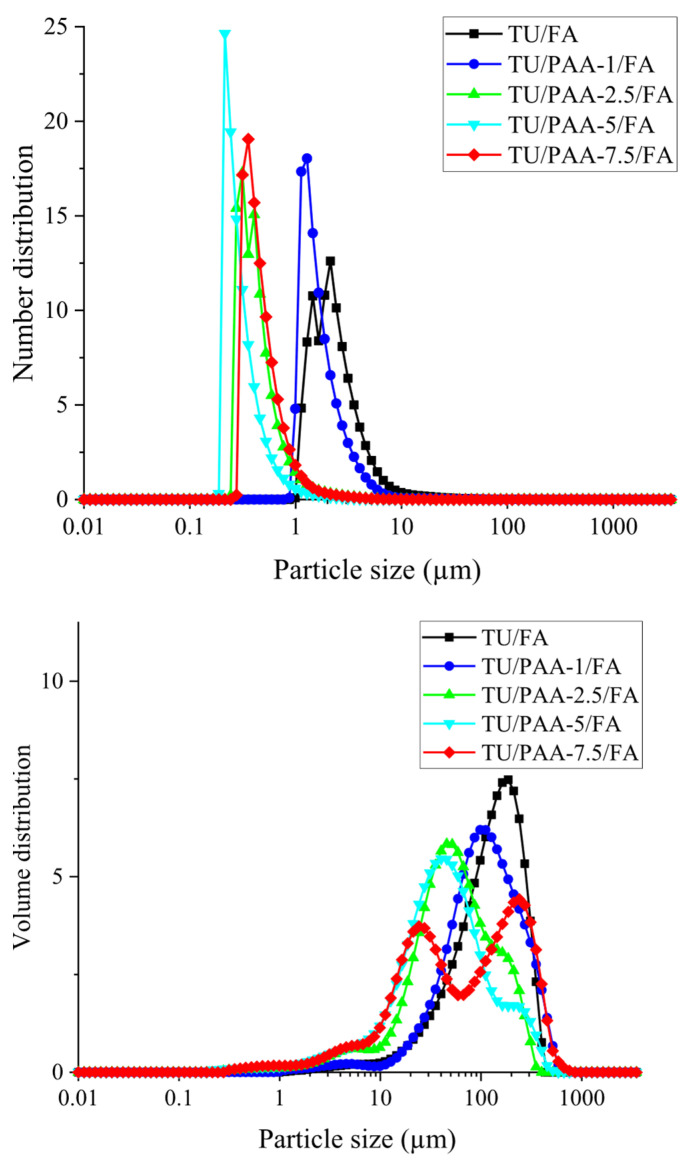
Particle size distributions (number and volume) of TU/FA and TU/PAA/FA materials.

On Figure 4 are reported the particle size distributions of TU/FA and TU/PAA/FA composites, represented in both number- and volume-based formats. Number-based distributions emphasize the primary particles, with numerous small particles strongly contributing to the signal, whereas volume-based distributions reflect mass contributions, often dominated by larger aggregates. The frequent discrepancy between the two distributions indicates the presence of aggregates: number distributions highlight the prevalence of fine particles, while volume distributions reveal the mass contribution of the largest particles [23].

For the reference TU/FA composite (without PAA), the main peak in the number distribution is located at 2.13 µm and 12.6% (Table 2), indicating a predominance of micrometric particles. The volume distribution shows a maximum at 186 µm (7.48%), corresponding to the contribution of large aggregates. Characteristic size parameters (Table 3) further support this observation: D10 = 1.66 µm, D50 = 2.228 µm, D90 = 4.58 µm for the number distribution, and D10 = 37.62 µm, D50 = 138.2 µm, D90 = 287.8 µm for the volume distribution. These results suggest that, in the absence of PAA, particle nucleation is less pronounced, particle growth is more extensive, and aggregation is more significant.

Pre-incorporation of PAA before FA addition alters the TU–FA polycondensation kinetics by promoting nucleation while limiting particle growth. PAA interacts with thiourea through hydrogen bonding and electrostatic interactions and increases local viscosity, influencing particle formation [24].

The effect of PAA adjunction is evidenced by a progressive shift of the peaks toward smaller sizes and a reduction in particle size parameters:

- TU/PAA-1/FA: Number peak at 1.28 µm (18.03%) and volume peak at 98.1 µm (6.2%). D50 values confirm this trend (number = 1.586 µm; volume = 114.2 µm). Large aggregates remain, but are smaller than in TU/FA.
- TU/PAA-2.5/FA: Number peak in the submicron range (0.31 µm—17.16%) and volume peak at 45.6 µm (5.83%). D50 values (number = 0.411 µm; volume = 61.46 µm) indicate efficient fine nucleation and limited growth.
- TU/PAA-5/FA: Number peak at 0.214 µm (24.64%) and volume peak at 40.1 µm (5.45%). D50 values (number = 0.2902 µm; volume = 44.42 µm) demonstrate the optimal effect of intermediate PAA content on particle size reduction and suppression of large aggregates.
- TU/PAA-7.5/FA: Number peak at 0.35 µm (19.05%) and bimodal volume distribution with maxima at 24.1 µm (3.73%) and 240 µm (4.42%). D50 values (number = 0.4524 µm; volume = 71.3 µm) reveal increased polydispersity at high PAA content, where fine nucleation and aggregation coexist.

It could be deduced that the pre-incorporation of PAA prior to formaldehyde addition significantly reduces primary particle size, with the strongest effect observed at intermediate PAA contents (2.5–5 mL). Low PAA content (1 mL) produces moderate reduction, whereas high PAA content (7.5 mL) increases polydispersity due to competition between nucleation and aggregation [25,26].

Two complementary approaches were employed to quantitatively illustrate these trends—Table 2 presents the coordinates of the main peaks in number- and volume-based distributions and Table 3 gathers the characteristic particle size parameters (D10, D50, D90, and D32)—providing a standardized quantitative description of particle size and enabling comparisons across formulations.

### 3.5. Surface Charge and Stability

The surface charge and colloidal stability of TU/FA and TU/PAA/FA composites were assessed through zeta potential and electrical conductivity measurements (Table 4). These parameters highlight the influence of pre-mixing poly(acrylic acid) (PAA) with thiourea (TH) prior to formaldehyde (FA) addition, as well as the effect of PAA content on electrostatic repulsion and particle dispersion.

For the TU/FA reference composite, the mean zeta potential was slightly negative (−2.92 ± 0.67 mV), indicating weak electrostatic repulsion and thus limited colloidal stability. The conductivity was also low (0.03 ± 0.002 mS/cm), reflecting a low concentration of free ions in the medium [27]. This weak surface charge is consistent with the presence of large aggregates observed in the particle size distributions.

Then, the incorporation of PAA markedly altered both zeta potential and conductivity, with effects depending on the PAA loading:

- TU/PAA-1/FA (low PAA content): The zeta potential shifted to a slightly positive value (1.39 ± 0.69 mV), while conductivity slightly decreased (0.02 ± 0.006 mS/cm). Partial adsorption of carboxylate groups from PAA on particle surfaces induced a weak positive charge, promoting finer dispersion and reducing aggregation through moderate electrostatic interactions.
- TU/PAA-2.5/FA (intermediate PAA): The zeta potential was close to neutrality (−1.18 ± 0.40 mV), whereas conductivity increased to 0.05 ± 0.001 mS/cm. These values indicate a balance between partial charge neutralization and ionization of PAA groups, favoring homogeneous dispersion and suppression of large aggregates [28].
- TU/PAA-5/FA and TU/PAA-7.5/FA (high PAA content): The zeta potential became strongly negative (−37.3 ± 1.66 mV and −41.4 ± 0.3 mV, respectively), accompanied by a substantial increase in conductivity (0.15 ± 0.002 and 0.15 ± 0.001 mS/cm). Enhanced adsorption and ionization of carboxylate groups generated strong electrostatic repulsion, which stabilized the particles and prevented aggregation even at higher viscosities [29].

These results demonstrate that pre-mixing PAA with thiourea before formaldehyde addition enables fine control of surface charge and colloidal stability. Intermediate to high PAA contents confer sufficient electrostatic repulsion to ensure stable dispersions, whereas low amounts provide only limited stabilization. These findings corroborate the particle size analyses reported in Section 3.4, confirming the pivotal role of PAA in modulating particle size and dispersion in TU–FA composites.

### 3.6. Thermal Stability

**Figure 5:**
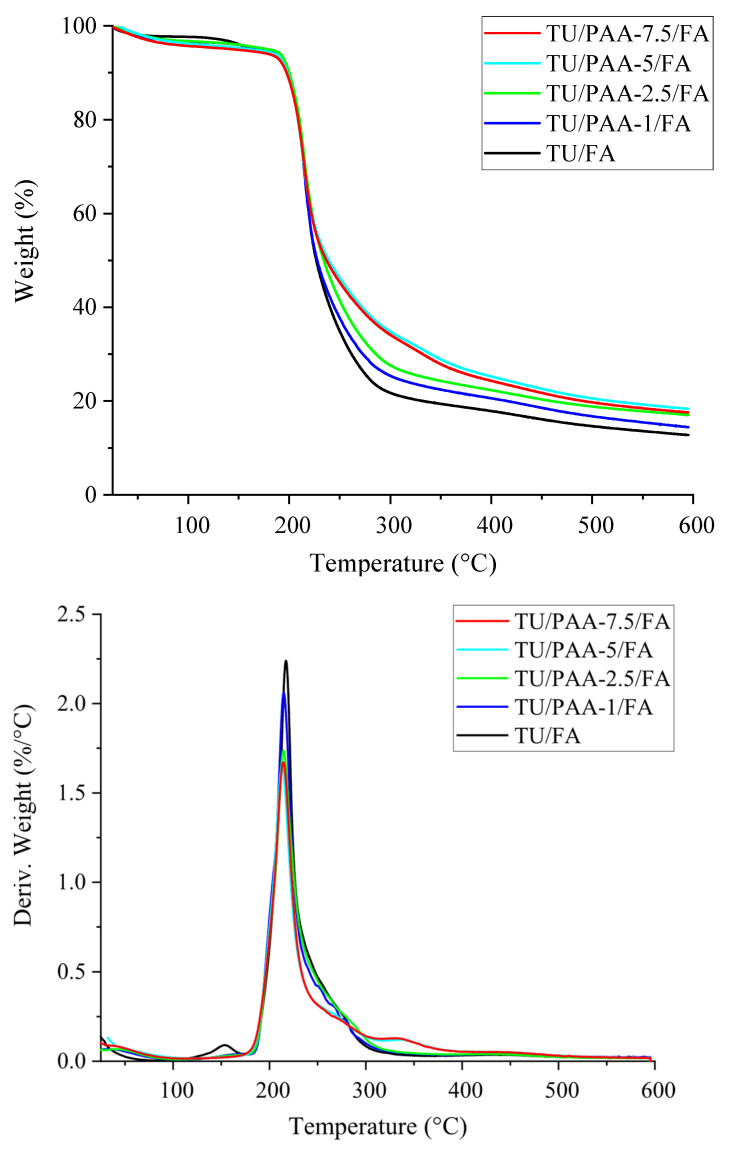
TGA and DTG curves of TU/FA and TU/PAA/FA composites.

The thermal degradation behavior of TU/FA and TU/PAA/FA composites was further assessed by thermogravimetric analysis (TGA) and derivative thermogravimetry (DTG). TGA and DTG curves are shown in Figure 5, and the corresponding parameters are summarized in Table 5.

The pre-incorporation of PAA with TH prior to FA crosslinking alters the thermal degradation profile. The DTG curve of TU/FA displays two degradation events, with a minor transition at 153.48 °C and a main degradation peak (Tmax) at 217.13 °C. The introduction of PAA eliminates the minor transition, suggesting a modification of the degradation mechanism. Moreover, PAA-containing composites exhibit superior thermal stability, as evidenced by a higher residual mass at 600 °C. TU/FA leaves 12.76% residue, while TU/PAA/FA composites retain between 14.43% and 18.35%. This higher char yield indicates improved resistance to thermal decomposition and more efficient char formation in the presence of PAA [30,31].

The effect of PAA concentration is also evident. Residual mass at 600 °C increases with PAA content, reaching a maximum of 18.35% for TU/PAA-5/FA. At higher PAA content (7.5%), the residue slightly decreases (17.60%), suggesting an optimal PAA loading for maximizing thermal stability. Tmax values for TU/PAA/FA composites are slightly lower than those of TU/FA, with differences ranging from 2.36 °C to 4.14 °C. The increase in residue correlates with a reduction in major weight loss, decreasing from 82.16% for TU/PAA-1/FA to 77.43% for TU/PAA-5/FA. These results confirm that PAA addition, particularly at 5% loading, substantially enhances the thermal stability of TU/PAA/FA composites [32,33].

Building on the comprehensive physicochemical characterization of the TU/PAA/FA composites, the next section explores their practical application as efficient adsorbents for removing crystal violet dye from aqueous solutions.

## 4. Application in Adsorption of Crystal Violet Dye

### 4.1. Effect of Experimental Parameters

**Figure 6:**
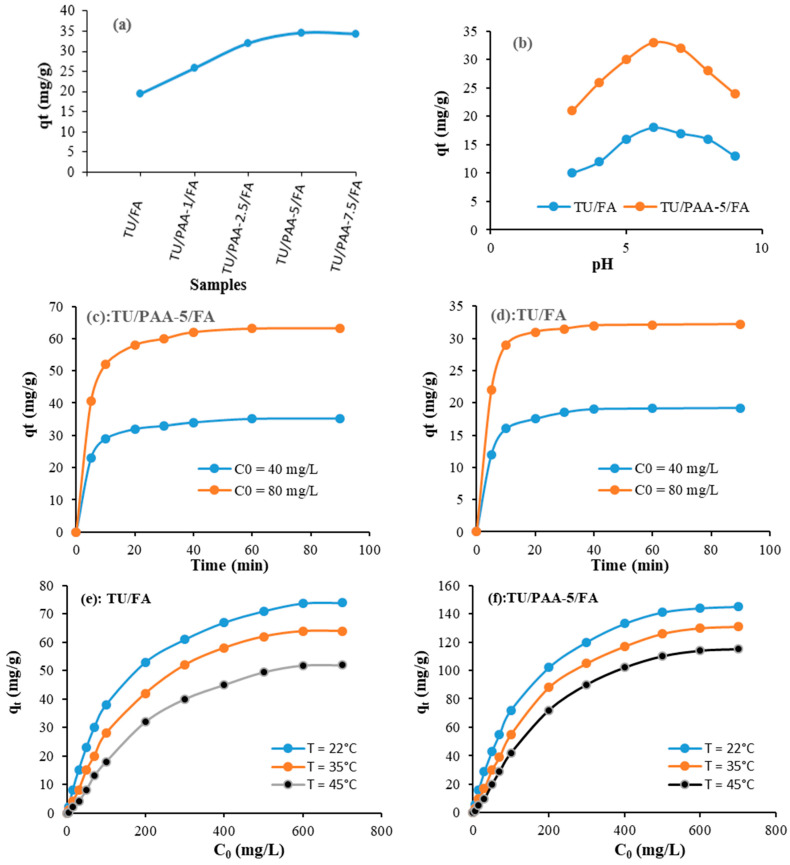
Effect of experimental conditions on the adsorption of crystal violet using TU/FA and TU/PAA-5/FA materials: (**a**) PAA dose change (C_0_ = 40 mg/L, T = 22 °C, time = 90 min), (**b**) pH change (C_0_ = 40 mg/L, T = 22 °C), (**c**,**d**) time change (pH = 6, T = 22 °C), and (**e**,**f**) initial crystal violet and temperature change (pH = 6, time = 90 min).

Figure 6a shows the progress of the adsorbed capacity of crystal violet using TU/FA and the different composites as adsorbents. As can be observed, the adsorption capacity increases with the increase in the incorporation of PAA dose and it achieves its maximum using the composite TU/PAA-5/FA. However, the adsorption capacity tends to decrease when the dose of the PAA is more than 5 mL. This result can be explained by the saturation of the adsorption sites. In the following experiments, the influences of pH, time, initial crystal violet concentration and temperature are discussed using the TU/PAA-5/FA and TU/FA composites as adsorbents. Figure 6b represents the effect of pH on the progress of the adsorption capacity. The results indicated that the adsorption was significantly influenced by the pH of the solution. In highly acidic conditions, the positive charge on the crystal violet molecules is stronger, but the studied adsorbent’s surface is more likely to be protonated and become positively charged. This trend leads to the occurrence of electrostatic repulsion between the dye and the adsorbent, resulting therefore in lower adsorption performance. As can be observed, the optimum adsorption capacity was reached when the pH was equal to 6. This can be justified by the fact that at this optimum pH condition, the adsorbent’s surface becomes more negatively charged, creating a stronger electrostatic attraction with the positively charged crystal violet molecules and consequently leading to better adsorption capacity.

Regarding the effect of time on the adsorption capacity (Figure 6c,d), the temporal patterns show that the crystal violet adsorption proceeds in multiple stages: an initial rapid uptake (most dye adsorbed within 10 min), followed by a slower approach to equilibrium by ~40 min.

The adsorption data (Figure 6) also clearly indicate that the adsorption capacity is significantly influenced by the initial crystal violet concentration and temperature (Figure 6e,f). It is observed that at optimum conditions, the adsorption quantities are equal to 145 mg/g and 74 mg/g for TU/PAA-5/FA and TU/FA, respectively. The good adsorption capacities achieved mainly in the case of the adsorbent TU/PAA-5/FA are justified by the addition of more carboxylic acid groups which could easily interact with the crystal violet. The adsorption capacity reached using the prepared composite TU/PAA-5/FA (145 mg/g) is considered important compared to other studied adsorbents in the literature (Table 6).

As is also displayed, the adsorption capacity depends on the temperature and it decreases with the increase in temperature. This exothermic effect may be due to the reduction in the interaction between crystal violet and the functional groups of the prepared adsorbents.

### 4.2. Kinetic Modeling

**Figure 7:**
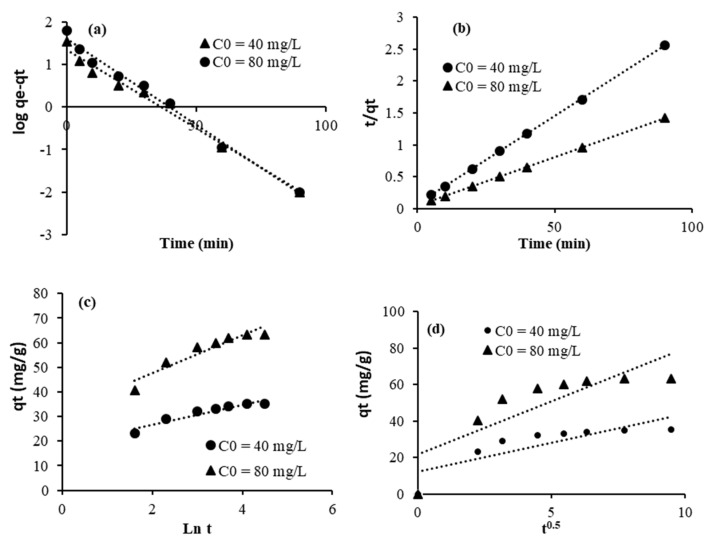
Linearized adsorption kinetic data for TU/FA: (**a**) pseudo-first-order, (**b**) pseudo-second-order, (**c**) Elovich, and (**d**) intraparticular diffusion.

**Figure 8:**
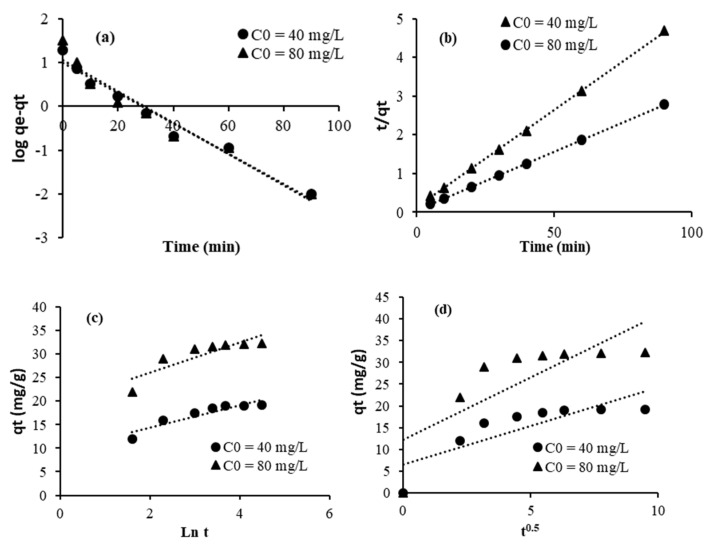
Linearized adsorption kinetic data for TU/PAA-5/FA: (**a**) pseudo-first-order, (**b**) pseudo-second-order, (**c**) Elovich, and (**d**) intraparticular diffusion.

The equations of pseudo-first-order (Figure 7a and Figure 8a), pseudo-second-order (Figure 7b and Figure 8b), Elovich (Figure 7c and Figure 8c), and intraparticular diffusion (Figure 7d and Figure 8d) are used to better understand the adsorption mechanism of crystal violet using the adsorbents TU/PAA-5/FA and TU/FA.

Kinetic parameters and regression coefficients are computed and given in Table 7 and Table 8. The pseudo-second-order model fits the experimental adsorption data remarkably well (R^2^ = 0.99). This conclusion is further supported by the strong correlation between the calculated capacities of crystal violet adsorbed onto the adsorbent surface and those determined theoretically. This observation implies that the adsorption mechanism is likely governed by chemisorption [39]. Indeed, the interaction between crystal violet and thiourea–formaldehyde is primarily an adsorption process driven by a combination of electrostatic forces (due to the cationic nature of crystal violet and potential charges on the adsorbent), hydrogen bonding, and pi–pi interactions between the aromatic rings of crystal violet and functional groups on the thiourea–formaldehyde complex. The incorporation of PAA into thiourea–formaldehyde, with its carboxylic groups, enhances the composite’s ability to adsorb the dye. Through electrostatic attraction between the positively charged crystal violet and the negatively charged carboxylate groups of PAA, this interaction can lead to the formation of a complex or aggregate between the dye and the polymer. Other factors like hydrogen bonding and van der Waals forces can also contribute to the binding.

We also observed that the plots do not pass through the origin, indicating that adsorption involves steps beyond intraparticle diffusion [40]. This statement means that the adsorption process involves multiple kinetic steps, not just diffusion within the pores. Thus, the overall adsorption is influenced by external mass transfer (or film diffusion), which is the movement of the adsorbate through the liquid film surrounding the adsorbent, and adsorption at the active sites, which is the chemical or physical binding on the surface.

The Elovich adsorption kinetic model is an empirical rate equation that states the adsorption energy rises in a linear relationship with surface coverage. The model assumes that adsorption occurs on localized sites, the interaction between adsorbed ions is present, and the concentration of adsorbate is considered constant [41]. Indeed, the low values of the regression coefficients observed within this model may suggest that the adsorption does not occur on localized sites.

### 4.3. Isotherm Modeling and Thermodynamic Studies

**Figure 9:**
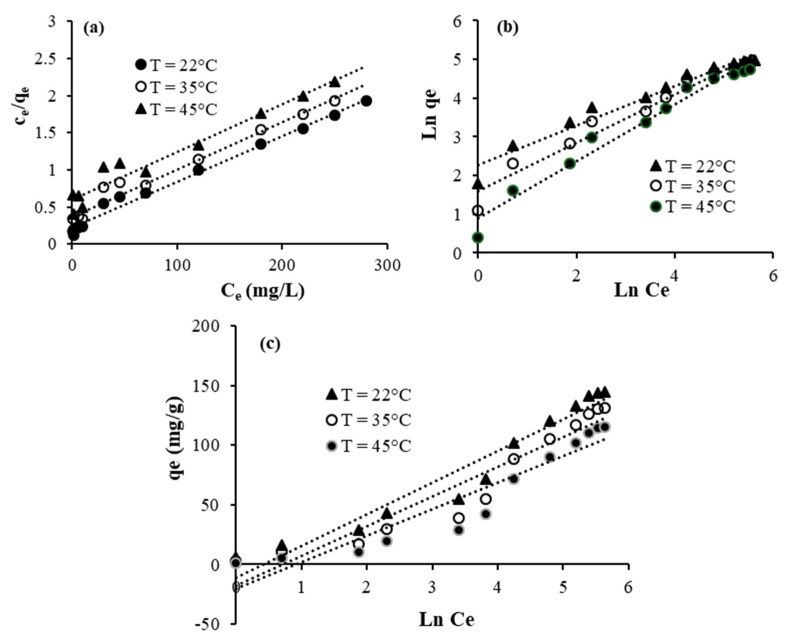
Linearized adsorption isotherms for TU/FA: (**a**) Langmuir, (**b**) Freundlich, (**c**) Temkin.

**Figure 10:**
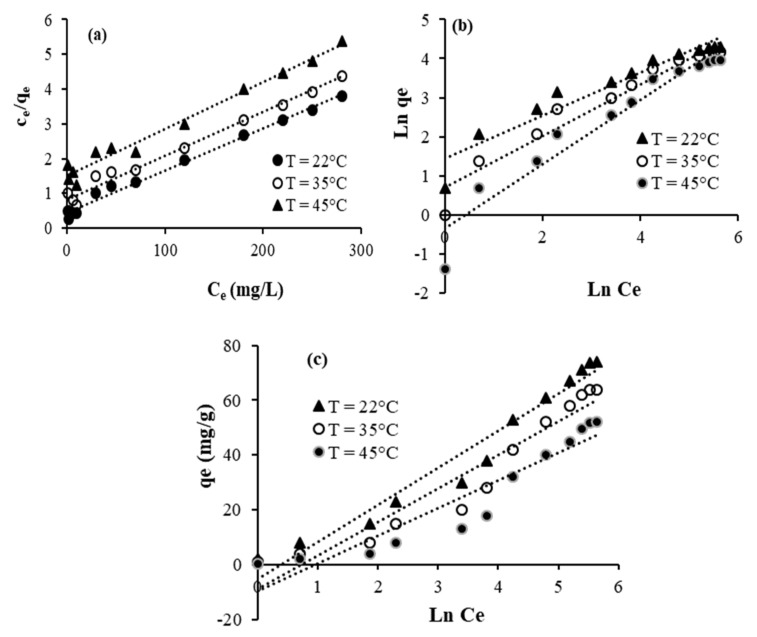
Linearized adsorption isotherm data for TU/PAA-5/FA: (**a**) Langmuir, (**b**) Freundlich, and (**c**) Temkin.

The isotherm equations of Langmuir (Figure 9a and Figure 10a), Freundlich (Figure 9b and Figure 10b), and Temkin (Figure 9c and Figure 10c) are used to better evaluate the interaction between crystal violet, TU/FA, and TU/PAA-5/FA. The isotherm constants and the regression coefficients are given in Table 7 and Table 8. As is revealed, the regression coefficients are higher than 0.97 in the case of the Langmuir isotherm. This supposes that the functional adsorption sites are homogeneously distributed onto the surface of TU/FA and TU/PAA-5/FA. The parameter n of the Freundlich model ranges from 1.36 to 2.09, which reveals that the adsorption of crystal violet is favorable at these conditions [42].

**Figure 11:**
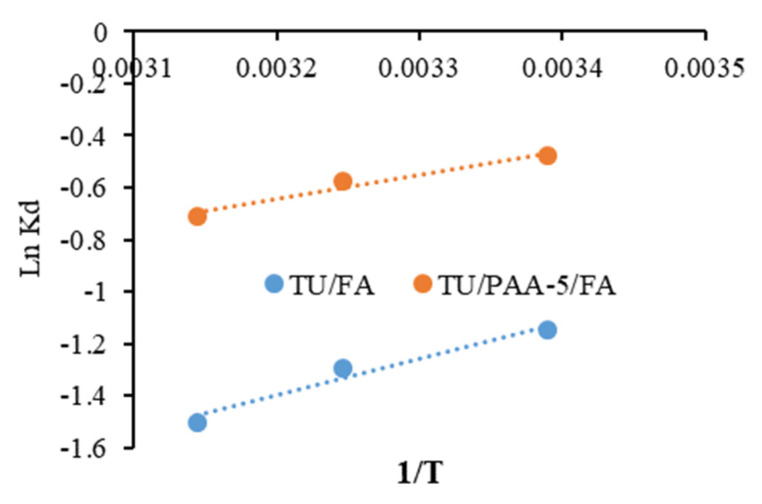
Plots of Ln kd vs. 1/T for TU/FA and TU/PAA-5/FA.

Figure 11 elucidates the plots of Logarithm Kd constants versus 1/T related to the adsorption of crystal violet using TU/FA and TU/PAA-5/FA. The enthalpy (ΔH°) and entropy ΔS° parameters are then determined. The negative ΔH° values indicate that the adsorption mechanism is exothermic. This complies well with the decrease in the adsorbed quantiles of crystal violet molecules with the increase in temperature. The negative ΔS° values designate that the disorder decreases during the adsorption and some structural changes occur. The positive ΔG° values mean that the adsorption of crystal violet at these conditions is non-spontaneous.

## 5. Conclusions

In this study, a series of thiourea–formaldehyde (TU/FA) and thiourea–poly(acrylic acid)–formaldehyde (TU/PAA/FA) composites was synthesized by pre-mixing PAA with thiourea prior to formaldehyde-driven polycondensation. The prior incorporation of PAA systematically altered the composite morphology, leading to more porous and open architectures, reduced primary particle size (optimal at 2.5–5 mL PAA), enhanced colloidal stability at higher PAA contents (zeta potential ≈ −37 and −41 mV for TU/PAA-5/FA and TU/PAA-7.5/FA, respectively), and improved thermal robustness.

These structure–property correlations demonstrate that TU/PAA/FA composites, particularly those prepared with intermediate PAA content, are promising candidates for the adsorption of cationic dyes, owing to their enhanced surface accessibility and favorable electrostatic interactions.

Furthermore, the adsorption performance toward crystal violet dye was quantitatively evaluated through kinetic, isotherm, and thermodynamic modeling. The TU/PAA-5/FA adsorbent exhibited superior adsorption capacity (145 mg/g) compared to TU/FA (74 mg/g), following pseudo-second-order kinetics and fitting well to the Langmuir model (R^2^ > 0.97). The exothermic and non-spontaneous nature of the process suggests the predominance of chemical adsorption and temperature-dependent interaction between dye molecules and functional groups.

These results confirm the potential of TU/PAA/FA materials as efficient adsorbents for cationic dye removal from aqueous media. Thus, future work could be focused on optimizing PAA loading, scaling up synthesis protocols for industrial applications, and exploring regeneration strategies for sustainable treatment of industrial dye effluents.

## Figures and Tables

**Table 1:** Composition and designation of TU/FA and TU/PAA/FA composites.

Sample	TU (g)/100 mL	PAA (mL)	FA (mL)
TU/FA	5.87	0.0	6
TU/PAA-1/FA	5.87	1.0	6
TU/PAA-2.5/FA	5.87	2.5	6
TU/PAA-5/FA	5.87	5	6
TU/PAA-7.5/FA	5.87	7.5	6

**Table 2:** Main peak coordinates in number and volume distributions.

Sample	Number Distribution		Volume Distribution	
	Peak (µm)	%	Peak1 (µm)	%
TU/FA	2.13	12.6	186	7.48
TU/PAA-1/FA	1.28	18.03	98.1	6.2
TU/PAA-2.5/FA	0.31	17.16	45.6	5.83
TU/PAA-5/FA	0.214	24.64	40.1	5.45
TU/PAA-7.5/FA	0.35	19.05	24.1	3.73

**Table 3:** Characteristic particle size parameters for number and volume distributions.

Sample	Number Distribution				Volume Distribution			
	D10 (µm)	D50 (µm)	D90 (µm)	D32 (µm)	D10 (µm)	D50 (µm)	D90 (µm)	D32 (µm)
TU/FA	1.66	2.228	4.58	1914	37.62	138,2	287.8	93.02
TU/PAA-1/FA	1.194	1.586	3.194	1.366	35.52	114.2	316.4	77.7
TU/PAA-2.5/FA	0.3242	0.411	0.7962	0.3634	14.90	61.46	164.8	37.62
TU/PAA-5/FA	0.225	0.2902	0.5508	0.2548	10.24	44.42	182.4	28.52
TU/PAA-7.5/FA	0.341	0.4524	0.8536	0.393	10.80	71.30	319.8	29.92

**Table 4:** ζ potential and electrical conductivity of synthetized composites.

Sample	ζ Potential ±SD (mV)	Conductivity ±SD (mS/cm)
TU/FA	−2.92 ± 0.67	0.03 ± 0.002
TU/PAA-1/FA	1.39 ± 0.69	0.02 ± 0.006
TU/PAA-2.5/FA	−1.18 ± 0.40	0.05 ± 0.001
TU/PAA-5/FA	−37.3 ± 1.66	0.15 ± 0.002
TU/PAA-7.5/FA	−41.4 ± 0.3	0.15 ± 0.001

**Table 5:** Thermogravimetric properties of TU/FA and TU/PAA/FA composites.

Sample	Initial Weight Loss (%)	Temp. Range (°C)	Tmax (°C)	Main Weight Loss (%)	Temp. Range (°C)	Tmax (°C)	Residue at 600 °C (%)
TU/FA	2.21	144–171	153.48	81.78	195- 314	217.13	12.76
TU/PAA-1/FA	3.40	145–176	159.01	82.16	197–322	214.77	14.43
TU/PAA-2.5/FA	3.295	145–173	156.55	79.65	199–331	214.54	17.05
TU/PAA-5/FA	4.219	146–174	154.17	77.43	197–321	212.99	18.35
TU/PAA-7.5/FA	4.464	147–173	155.71	77.93	198–342	214.47	17.60

**Table 6:** A comparison of the adsorption capacities of crystal violet using some adsorbents published in the literature.

Adsorbent	Maximum Adsorption Capacity (mg/g) at Optimum Conditions	Reference
TU/PAA-5/FA composite	145	Current work
Ethylene diamine/reduced graphene oxide/cellulose composite	260	[34]
Tire rubber and polyurethane foam composite	20.92	[35]
Granular biopolymer–silica/clay composites	208.9	[36]
Chitosan aniline composite	100.6	[37]
Fe3O4–graphene–biochar composite	157.3	[38]

**Table 7:** Kinetic data and Langmuir, Freundlich, and Temkin parameters for the adsorption of crystal violet onto TU/FA.

Kinetic equations	Constants	Crystal violet concentration (mg/L)		Isotherms	Parameters	Temperature (°C)		
Pseudo-first-order		40	80			22	35	45
	K_1_ (min^−1^)	0.034	0.036		q_L_ (mg·g^−1^)	83.33	78.74	74.07
	q (mg·g^−1^)	9.54	11.30	Langmuir	K_L_ (L·g^−1^)	0.026	0.016	0.01
	R^2^	0.97	0.94		R^2^	0.99	0.98	0.98
Pseudo-second-order	K_2_	0.02	0.019	Thermodynamic parameters	ΔH° (KJ mol^−1^)	−11.7		
	q (mg·g^−1^)	20.00	32.89		ΔS° (J mol^−1^)	−49		
	R^2^	0.99	0.99		ΔG° (KJ mol^−1^)	2.76	3.4	3.89
				Freundlich	K_F_ (L.g^−1^)	27.54	5.01	7.87
Elovich	*α* (mg·g^−1^·min^−1^)	132.69	1623.4		*n*	1.82	1.51	1.35
	*β* (mg·g^−1^·min^−1^)	0.42	0.32		R^2^	0.92	0.94	0.93
	R^2^	0.86	0.76	Temkin	b_T_ (J.mol^−1^)	180.87	208.52	261.50
Intraparticular diffusion	K (mg·g^1^·min^1/2^)	1.78	2.86		A_T_ (L.g^−1^)	1.49	2.09	2.63
	R^2^	0.69	0.62		R^2^	0.95	0.92	0.89

**Table 8:** Summarized kinetic data and Langmuir, Freundlich, and Temkin parameters for the adsorption of crystal violet onto TU/PAA-5/FA.

Kinetic equations	Constants	Crystal violet concentration (mg/L)		Isotherms	Parameters	Temperature (°C)		
Pseudo-first-order		40	80			22	35	45
	K_1_ (min^−1^)	0.037	0.041		q_L_ (mg·g^−1^)	161.29	156.25	156.25
	q (mg·g^−1^)	22.86	41.49	Langmuir	K_L_ (L·g^−1^)	0.027	0.018	0.011
	R^2^	0.98	0.99		R^2^	0.98	0.97	0.97
Pseudo-second-order	K_2_	0.01	0.006	Thermodynamic parameters	ΔH° (KJ mol^−1^)	−7.73		
	q (mg·g^−1^)	36.36	65.35		ΔS° (J mol^−1^)	−30.088		
	R^2^	0.99	0.99		ΔG° (KJ mol^−1^)	1.14	1.53	1.83
				Freundlich	K_F_ (L·g^−1^)	184.07	39.81	7.80
Elovich	*α* (mg·g^−1^·min^−1^)	361.94	513.96		n	1.96	1.61	1.36
	*β* (mg·g^−1^·min^−1^)	0.24	0.13		R^2^	0.95	0.96	0.97
	R^2^	0.91	0.89	Temkin	b_T_ (J·mol^−1^)	92.37	102.96	118.66
Intraparticular diffusion	K (mg·g^1^·min^1/2^)	3.21	5.83		A_T_ (L·g^−1^)	1.51	2.03	2.49
	R^2^	0.69	0.69		R^2^	0.95	0.92	0.89

## Data Availability

The original contributions presented in this study are included in the article. Further inquiries can be directed to the corresponding authors.
